# Angiogenesis and epidermal growth factor receptor inhibitors in non-small cell lung cancer

**DOI:** 10.37349/etat.2020.00008

**Published:** 2020-04-28

**Authors:** Giuliano Palumbo, Giovanna Esposito, Guido Carillio, Anna Manzo, Agnese Montanino, Vincenzo Sforza, Raffaele Costanzo, Claudia Sandomenico, Carmine La Manna, Nicola Martucci, Antonello La Rocca, Giuseppe De Luca, Maria Carmela Piccirillo, Rossella De Cecio, Francesco Perrone, Giuseppe Totaro, Paolo Muto, Carmine Picone, Nicola Normanno, Alessandro Morabito

**Affiliations:** 1 Department of Oncology, Ospedale S. Maria della Pietà, Casoria, 80026 Napoli, Italy; 2 Thoracic Medical Oncology, Istituto Nazionale Tumori “Fondazione G. Pascale”-IRCCS, 80131 Napoli, Italy; 3 Department of Oncology & Hematology, Azienda Ospedaliera Pugliese-Ciaccio, 88100 Catanzaro, Italy; 4 Thoracic Surgery, Istituto Nazionale Tumori “Fondazione G. Pascale”-IRCCS, 80131 Naples, Italy; 5 Clinical Trials Unit, Istituto Nazionale Tumori “Fondazione G. Pascale”-IRCCS, 80131 Naples, Italy; 6 Pathology, Istituto Nazionale Tumori “Fondazione G. Pascale”-IRCCS, 80131 Naples, Italy; 7 Radiotherapy, Istituto Nazionale Tumori “Fondazione G. Pascale”, IRCCS, 80131 Naples, Italy; 8 Radiology, Istituto Nazionale Tumori “Fondazione G. Pascale”- IRCCS, 80131 Naples, Italy; 9 Cellular Biology and Biotherapy, Istituto Nazionale Tumori “Fondazione G. Pascale”-IRCCS, 80131 Naples, Italy; University of Southampton, UK

**Keywords:** Lung cancer, angiogenesis, tyrosine kinase inhibitor, erlotinib, bevacizumab

## Abstract

Several preclinical studies suggested a potential benefit from combined treatment with inhibitors of epidermal growth factor receptor (EGFR) and angiogenesis, both effective in patients with advanced non-small-cell lung cancer (NSCLC). In pretreated patients with advanced EGFR wild type NSCLC, bevacizumab plus erlotinib improved progression-free survival as second-line therapy in the BeTa study and as maintenance therapy in the ATLAS trial, although the benefit was modest and did not translate into an advantage in overall survival. Disappointing results were reported with oral VEGF inhibitors plus erlotinib in pretreated patients with EGFR wild type NSCLC. On the contrary, erlotinib plus bevacizumab or ramucirumab showed a clinically relevant improvement of progression-free survival in naïve patients with EGFR mutations, leading to the approval of these two regimens as first-line treatment of NSCLC patients with EGFR mutant tumors. Several clinical studies are evaluating the feasibility and activity of osimertinib plus bevacizumab or ramucirumab. However, limits that could affect its use in clinical practice are the need of an intravenous infusion for angiogenesis inhibitors, the increased incidence of treatment associated adverse events, the exclusion of patients with tumors located in central position or at risk of hemorrhage. The identification of predictive biomarkers is an important goal of research to optimize the combined use of these agents.

## Introduction

Angiogenesis is a crucial process for the development of almost all tumors, including non-small-cell lung cancer (NSCLC). The acquisition of the ability to induce the formation of blood vessels, also known as the angiogenic switch, is important to ensure tumor growth. In this respect, the vascular endothelial growth factor (VEGF) family plays an important role in this process. VEGF family consists of five members/ligands (VEGF-A, VEGF-B, VEGF-C, VEGF-D, and placental growth factor) and three receptors. VEGF-A (VEGF) is the most important factor involved in angiogenesis [1–3]. The three VEGF receptors include Flt-1 (VEGFR-1), KDR (VEGFR-2), and Flt-4 (VEGFR-3), with intrinsic tyrosine kinase activity induced by ligand binding and receptor dimerization [4]. In tumors, the deregulation of VEGF signaling results in blood vessel creation with anarchic organization, promotion of tumor growth and tumor cells metastasis.

Three angiogenesis inhibitors are approved in NSCLC: bevacuzimab, ramucirumab and nintedanib. Bevacizumab is a monoclonal antibody that targets VEGF. It has been approved in combination with platinum-based chemotherapy for untreated patients with unresectable, locally advanced, recurrent or metastatic non-squamous NSCLC, according to the results of two randomized phase III studies: ECOG 4599 and AVAiL [5–6]. These trials included patients with non-squamous histology and without major hemorrhage risk factors. Ramucirumab is a human monoclonal immunoglobuline G1 antibody targeting VEGFR-2 and interfering with activity of different VEGF isoforms, including VEGF-A. Ramucirumab is indicated in combination with docetaxel in metastatic NSCLC with disease progression on or after platinum-based chemotherapy. Patients with EGFR or anaplastic lymphoma kinase (ALK) alterations should have disease progression on FDA-approved therapy for these targets prior to receiving ramucirumab [7]. Nintedanib is an oral triple angiokinase inhibitor targeting the angiogenic pathways mediated by VEGFR1-3, fibroblast growth factor receptors (FGFR) 1–3, and platelet-derived growth factor receptors (PDGFR): nintedanib plus docetaxel have been approved for patients with locally advanced/metastatic NSCLC of adenocarcinoma tumor histology after first-line therapy [8].

The epidermal growth factor receptor HER-1/EGFR besides being mutated may be overexpressed in several tumors, including NSCLC. EGFR overexpression results in activation of tyrosine kinase activity, triggering intracellular pathways resulting in apoptosis blocking, cancer-cell proliferation, invasion, metastasis and neovascularization [9]. Different EGFR tyrosine kinase inhibitors (TKIs) can be used in advanced NSCLC: gefitinib, erlotinib, afatinib, dacomitinib, and osimertinib are indicated for patients with EGFR activating mutations, while erlotinib has been approved also for patients with EGFR wild type (WT) tumors pretreated with first- or second-line therapy [10–13].

**Figure 1.**
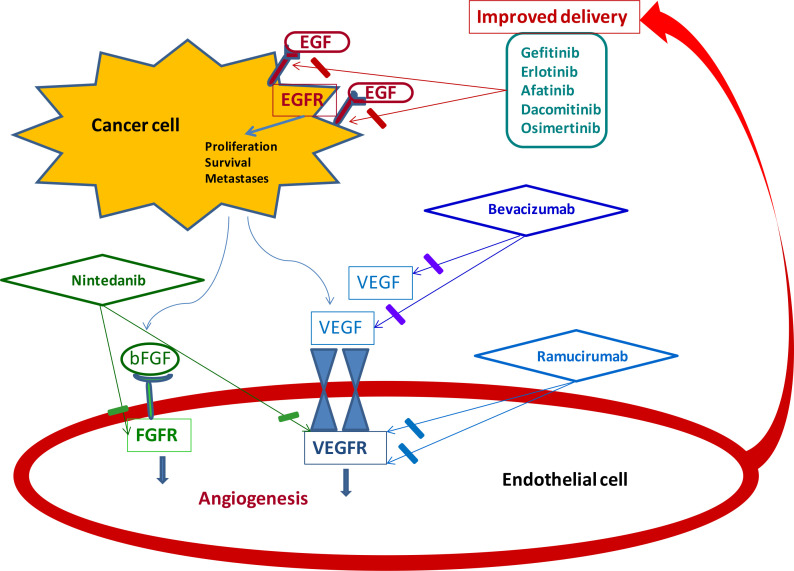
Synergistic effects with dual blockade of angiogenesis and EGFR. bFGF: beta fibroblast growth factor

VEGF and EGFR share common downstream signaling pathways and preclinical data showed that EGFR stimulation could increase VEGF expression, providing a potential hypothesis that a dual blockade of these molecular targets may have synergistic effects (Figure 1) [14–16].

This review focuses on the dual inhibition of angiogenesis and EGFR in NSCLC, discussing the results of clinical studies and the most important issues that may limit this combination in the clinical practice.

## Angiogenesis plus EGFR inhibitors in “EGFR wild type” patients

### Bevacizumab plus erlotinib

A number of studies have evaluated bevacizumab plus erlotinib in advanced NSCLC patients with EGFR wild type tumors (Table 1). The first clinical studies with erlotinib plus bevacizumab were conducted by Herbst et al. [17–18], in cisplatin-refractory NSCLC. A phase I/II study evaluated the dose limiting toxicity (DLT) and activity of bevacizumab plus erlotinib in 40 patients with non-squamous NSCLC, in progression after a platinum first-line treatment, regardless of EGFR status [17]. No DLTs were reported: the recommended treatment was bevacizumab 15 mg/kg every three weeks as intravenous infusion and erlotinib 150 mg/day orally. The most frequent adverse events (AEs) were rash (85% of patients), diarrhea (65%) and infection (29%). Overall, 20% of patients had a partial response (PR), while 65% had stable disease. Notably, only nine patients had enough material to proceed for an EGFR mutational analysis and in two cases a mutation was found. Thereafter, in a randomized phase II study [18], 120 patients with non-squamous NSCLC in progression after a platinum treatment were treated with bevacizumab plus erlotinib, or bevacizumab plus docetaxel/pemetrexed or chemotherapy alone. Bevacizumab plus erlotinib was better tolerated than bevacizumab plus chemotherapy: serious AEs were reported in 55%, 41% and 33% of patients in chemotherapy, bevacizumab-chemotherapy and bevacizumab-erlotinib arm, respectively. Fatigue, nausea and vomiting were commonly reported in all 3 arms. Median progression-free survival (PFS) was 4.8, 4.4 and 3 months for bevacizumab plus chemotherapy, bevacizumab plus erlotinib and chemotherapy alone, respectively. Median overall survival (OS) was 12.6, 13.7 and 8.6 months for the bevacizumab plus chemotherapy, bevacizumab plus erlotinib and chemotherapy alone arm, respectively. The risk of progression or death was 0.66 [95% confidence interval (CI): 0.38–1.16] and 0.72 (95% CI: 0.42–1.23) for patients treated with bevacizumab plus chemotherapy or bevacizumab plus erlotinib against chemotherapy alone arm, respectively. No clinically meaningful differences were found between bevacizumab-chemotherapy and bevacizumab-erlotinib arms. Interestingly, one complete response was observed in a patient with an activating EGFR mutation treated with bevacizumab-erlotinib. These promising results prompted a randomized, double-blind, phase III clinical study (BeTa Trial) to compare bevacizumab plus erlotinib with erlotinib in 636 patients with advanced NSCLC in progression after a first-line treatment [19]. Unfortunately, the addition of bevacizumab to erlotinib did not improve the OS that was 9.3 months for bevacizumab-erlotinib *versus* 9.2 months for erlotinib group, respectively (*P* = 0.7583). Furthermore, grade 5 AEs were 6% in the bevacizumab group, including two arterial thromboembolic events, and 4% in the erlotinib group. In 30 patients with EGFR-mutant tumors, the hazard ratio (HR) for OS was lower in the bevacizumab than in the control group (HR 0.44, 95% CI: 0.11–1.67). A phase III trial compared second-line erlotinib plus bevacizumab and panitumumab *versus* erlotinib in 297 metastatic EGFR unselected NSCLC Chinese patients, pretreated with platinum-based chemotherapy [20]. The trial demonstrated an improvement in median PFS (4.6 *versus* 1.9 months, in experimental and erlotinib arm, respectively, *P* = 0.003) and in median OS (10.4 *versus* 8.9 months, in experimental and erlotinib arm, respectively, *P* = 0.031). However, severe diarrhea and rash were more common in the experimental than in the control arm (24% and 26% *versus* 8.5% and 8.5%, respectively). The safety and activity of bevacizumab plus erlotinib in pretreated non-squamous NSCLC patients with asymptomatic untreated brain metastases were evaluated in a phase II study (BRAIN trial) [21]. However, only 24 patients were enrolled and results may have only an exploratory value: response rate (RR) was 12.5%, 6-month PFS rate 57.2% and median OS 12 months. AEs were similar to those reported with previous trials of bevacizumab; one patient had grade 1 intracranial hemorrhage. Finally, the ATLAS trial was a randomized phase III study that evaluated the efficacy of erlotinib plus bevacizumab *versus* bevacizumab plus placebo as maintenance therapy in 745 patients with metastatic non-squamous NSCLC, without progression after four cycles of first-line chemotherapy plus bevacizumab [22]. The bevacizumab-erlotinib combination improved median PFS (4.8 *versus* 3.7 months, HR 0.71, 95% CI: 0.58–0.86), but not OS (14.4 in bevacizumab-erlotinib *versus* 13.3 months in the bevacizumab arm, respectively, HR 0.92, 95% CI: 0.7–1.21). Moreover, adverse events, severe AEs (mainly rash and diarrhea), and AEs leading to erlotinib/placebo discontinuation were observed more frequently with bevacizumab-erlotinib combination. On this basis, due to the modest benefit in PFS, the lack of benefit in OS and the worst toxicity profile, erlotinib plus bevacizumab didn’t become a new standard of care as second-line or maintenance therapy.

**Table 1.** Bevacizumab and erlotinib in EGFR wild type NSCLC patients

**Author**	**Phase**	**Line of treatment**	**Pts**	**Treatment**	**Response rate(%)**	**Progression-free survival (months)**	**Overall survival (months)**
Herbst et al. [17]	I/II	Second line	40	Erlotinib + Bevacizumab	20 95% CI: 7.6-32.4%	7.0; 1-year PFS = 38.0%, 95% CI: 24.3-59.6%	12.6; 1-year OS = 54.2%, 95% CI: 40.0%-73.4%
Herbst et al. [18]	II	Second line	120	Bevacizumab + CT* *vs.* bevacizumab + erlotinib *vs.* CT*	12.5 *vs.* 17.9 *vs.* 12.2	4.8 *vs.* 4.4 *vs.* 3.0 HR: 0.66, 95% CI: 0.38-1.16 HR: 0.72, 95% CI: 0.42-1.23	12.6 *vs.* 13.7 *vs.* 8.6 HR: 0.71, 95% CI: 0.41-1.21 HR: 0.78, 95% CI: 0.46-1.31
Herbst et al. BeTa trial [19]	III	Second line	636	Erlotinib + Bevacizumab *vs.* Erlotinib + placebo	14 *vs.* 7	3.4 *vs.*1.7 HR: 0.62, 95% CI: 0.52-0.75	9.3 *vs.* 9.2 HR: 0.97, 95% CI: 0.80-1.18, *P =* 0.7583
Wang et al. [20]	III	Second line	297	Erlotinib + Bevacizumab + Panitumumab *vs.* Erlotinib + placebo	38 *vs.*15 *P =* 0.014	4.6 *vs.* 1.9 *P =* 0.003	10.4 *vs.* 8.9 *P =* 0.031
Besse et al. BRAIN trial [21]	II	Second line	24	Erlotinib + Bevacizumab	12.5 95% CI: 2.7-32.4	6.3 95% CI: 3.0-8.4	12.0 95% CI: 8.9-20.2
Johnson et al. ATLAS trial [22]	III	Maintenance after I line	745	Erlotinib + Bevacizumab *vs.* Bevacizumab alone	-	4.8 *vs.* 3.7, HR: 0.71, 95% CI: 0.58-0.86 *P* < 0.001	14.4 *vs.* 13.3 HR: 0.92, 95% CI: 0.7-1.21 *P =* 0.5341
Dingemans et al. [23]	II	First line	47	Erlotinib + Bevacizumab	25	3.8 (95% CI: 2.3-5.4)	6.9 (95% CI: I 5.5-8.4)
Zappa et al. trial SAKK 19/05 [24]	II	First line	101	Erlotinib + Bevacizumab	17.8	4.1 (95% CI: 2.9-5.5)	14.1 (95% CI: 10.7-19.0)
Ciuleanu et al. TASK trial [25]	II	First line	200	Erlotinib + Bevacizumab *vs.* Chemo + Bevacizumab	23.8 *vs.* 34.4, *P =* 0.19	18.4 *vs.* 25 weeks HR 2.05, 95% CI: 1.11-3.77	16.4 *vs.* n.r HR:1.24, 95% CI: 0.75-2.05
Thomas et al. INNOVATIONS [26]	II	First line	224	Erlotinib + Bevacizumab *vs.* Cisplatin + Gemcitabine + Bevacizumab	12 *vs.* 36, *P* < 0.0001	3.5 *vs.* 6.9 HR: 1.85; 95% CI:1.39-2.45 *P* < 0.0001	12.6 *vs.*17.8 HR: 1.41; 95% CI: 1.01-1.97 *P =* 0.04

* Docetaxel or erlotinib; n.r: not reached

Several studies have also been conducted with bevacizumab plus erlotinib as first-line therapy in advanced NSCLC patients unselected for EGFR status. A phase II trial in 47 patients naïve for any treatment met the primary end-point that was the non-progression-rate (NPR) at 6 weeks, with a 75% NPR at 6 weeks [23]. However, OS was disappointing (6.9 months) and some patients experienced serious AEs, including a kidney thrombosis, a sigmoid colon perforation and thromboembolic events. The SAKK 19/05 trial evaluated the activity and feasibility of bevacizumab plus erlotinib followed at disease progression by platinum-based chemotherapy in 101 metastatic non-squamous NSCLC patients [24]. This trial did not meet the primary end-point, with a disease stabilization rate at twelve weeks of 54.5% (lower than expected). Moreover, only 63% of patients proceeded to second-line chemotherapy. A disease stabilization rate of 83.3% was observed in EGFR mutated patients (13.8% of cases). The most common AEs with the combination treatment were acne, fatigue and diarrhea. The TASK trial compared erlotinib plus bevacizumab *versus* chemotherapy plus bevacizumab in naïve patients with metastatic non-squamous NSCLC, unselected for EGFR [25]. At an interim analysis, PFS was significantly worse with bevacizumab plus erlotinib compared with bevacizumab plus chemotherapy arm (median PFS 18.4 *versus* 25 weeks, respectively; HR 2.05, 95% CI: 1.11–3.77). Moreover, a subgroup analysis showed no benefit with erlotinib treatment also in patients with EGFR positive cancers, probably due to the small sample size. AEs occurred in 84.1% and 82% in the experimental and control group, respectively: the most frequent AEs in the bevacizumab-chemotherapy arm were nausea and neutropenia, while in bevacizumab-erlotinib arm diarrhea and cutaneous rash. A randomized phase II study (INNOVATIONS trial) compared erlotinib plus bevacizumab (EB) against cisplatin, gemcitabine and bevacizumab (PGB) in 224 non-squamous, unselected, metastatic NSCLC patients as first-line therapy [26]. The combination of chemotherapy plus bevacizumab showed superior OS (HR 1.41, *P* = 0.0409), PFS (HR 1.85, *P* < 0.0001) and RR (36% *versus* 12%, *P* < 0.0001) compared with erlotinib plus bevacizumab. In patients with EGFR activating mutations (20%), the response rate was 25% *versus* 17% for for EB *versus* PGB, respectively. No difference in PFS (4.2 *versus* 4.6 months for EB and PGB, respectively) and only a positive trend for OS (17.0 *versus* 10.0 months, for EB and PGB, respectively; HR 0.45, 95% CI: 0.18–1.16, *P* = 0.092) were observed. The most common AEs in EB group were diarrhea and skin rash, while in the control group were thrombocytopenia and thromboembolic events.

### Oral VEGF inhibitors and erlotinib

Erlotinib has been combined with oral VEGF inhibitors, including sorafenib and sunitinib in NSCLC patients (Table 2). Sorafenib is a small molecule targeting VEGFR-2 and VEGFR-3, rapidly accelerated fibrosarcoma (Raf), rearranged during transfection (RET), tyrosine-protein kinase KIT, FMS-like tyrosine kinase 3 (FLT-3), and PDGFR-β, with limited anti-tumor activity in NSCLC when used as a single-agent [27–28]. A phase II study compared erlotinib plus sorafenib *versus* erlotinib plus placebo in 168 pretreated NSCLC patients [29]. In both treatment arms the majority of patients had a non-squamous histology, was female, and already received bevacizumab in a prior line. An EGFR and kirsten rat sarcoma (KRAS) mutation test was done in 65% of the patients and most of them were WT. The primary endpoints [overall response rate (ORR) and PFS] were similar in the two groups. Sixty-seven patients with EGFR wild-type tumors had a median PFS of 3.38 *versus* 1.77 months (*P* = 0.018) and a median OS of 8 *versus* 4.5 months (*P* = 0.019) with erlotinib plus sorafenib *versus* erlotinib plus placebo, respectively. These data suggest a potential benefit for the combination in EGFR-WT tumors, more dependent on other signalling pathways, such as PDGFR, VEGFR or Raf inhibited by sorafenib. Sunitinib is a kinase inhibitor targeting VEGFR-1, -2 and -3, PDGFR alpha and beta, stem-cell factor receptor (KIT), FLT3 and RET that has demonstrated antitumor activity as a single agent in phase II studies, in patients with refractory NSCLC, with RR of 2.1–11.1% [30–31]. In a phase III study, erlotinib 150 mg and blinded sunitinib 37.5 mg or placebo were administered orally once daily every 4-week in patients with recurrent NSCLC [32]. Nine hundred-sixty patients were randomized; the majority of them had a diagnosis of lung adenocarcinoma, with unknown EGFR status. OS (primary endpoint) was similar between the two treatment arms (9.0 *versus* 8.5 months in erlotinib plus sunitib *versus* erlotinib, respectively). Patients treated in the combination arm had a modest benefit in terms of PFS (3.6 *versus* 2.0 months, HR 0.807, 95% CI: 0.695–0.937, *P* = 0.0023), and ORR (10.6% *versus* 6.9%, *P* = 0.0471), respectively. Negative results were also reported by a phase II study that compared erlotinib plus sunitinib *versus* erlotinib plus placebo, in patients with metastatic NSCLC pretreated with one or two prior chemotherapy lines [33]. The combination of sunitinib and erlotinib did not improve PFS (2.8 *versus* 2.0, HR 0.898, *P* = 0.321), OS (8.2 *versus* 7.6 months, HR 1.066, *P* = 0.617) or objective response rates (4.6% *versus* 3.0%, respectively). A biomarker analysis showed a better PFS for patients with low-tumor PDGFR-*alpha* RNA in the combination arm, suggesting the relevance of tumor vascular microenvironment when combining angiogenesis and EGFR inhibitors. In both trials rash, diarrhea, fatigue and nausea were more frequent with the combined treatment.

**Table 2.** Oral VEGFR inhibitors and erlotinib in EGFR wild type NSCLC patients

**Author**	**Phase**	**Line of Treatment**	**Patients**	**Treatment**	**Response rate (%)**	**Progression-free survival (months)**	**Overall survival (months)**
Spigel et al. [29]	II	Second line	168	Sorafenib + erlotinib *vs.* placebo + erlotinib	8 *vs.* 11, *P* = 0.555	3.38 *vs.* 1.94, HR: 0.86; 95% CI: 0.60-1.22 *P* = 0.196	7.62 *vs.* 7.23, HR: 0.89; 95% CI: 0.59-1.34 *P* = 0.290
Scagliotti et al. [32]	III	Second line	960	Sunitinib + erlotinib *vs.* placebo + erlotinib	10.6 *vs.* 6.9, *P* = 0.0471	3.6 *vs.* 2.0 HR: 0.807; 95% CI: 0.695-0.937 *P* = 0.0023	9.0 *vs.* 8.5, HR: 0.922; 95% CI: 0.797-1.067 *P* = 0.1388
Groen et al. [33]	II	Second line	132	Sunitinib + erlotinib *vs.* placebo + erlotinib	4.6 *vs.* 3.0, *P* = 0.624	2.8 *vs.* 2.0 HR: 0.898; 80% CI: 0.671-1.203 *P* = 0.321	8.2 *vs.* 7.6 HR: 1.066; 95% CI: 0.705-1.612 *P* = 0.617

### Angiogenesis plus EGFR inhibitors in “EGFR mutated” patients

Several clinical trials evaluated the activity of the dual inhibition of angiogenesis and EGFR in NSCLC patients with EGFR mutation, including combination of erlotinib plus bevacizumab, gefitinib plus bevacizumab, afatinib plus bevacizumab, erlotinib plus ramucirumab (Table 3).

**Table 3.** VEGF and EGFR TKIs in EGFR mutated NSCLC patients

**Author**	**Phase**	**Line of Treatment**	**Patients**	**Treatment**	**Response rate (%)**	**Progression-free survival (months)**	**Overall survival (months)**
Seto et al. JO25567 trial [34, 35]	II	First	154	Erlotinib + bevacizumab *vs.* erlotinib	69 *vs.* 64, *P* = 0.49	16 *vs.* 9.7, HR: 0.54; 95% CI: 0.36-0.79 *P* = 0.0015	47 *vs.* 47.4, HR: 0.81; 95% CI: 0.53-1.23 *P* = 0.3267
Saito et al. NEJ026 trial [36]	III	First	228	Erlotinib + bevacizumab *vs.* erlotinib	72 *vs.* 66, *P* = 0.31	16.9 *vs.*13.3, HR: 0.605; 95% CI: 0.417-0877; *P* = 0.016.	n.r.
Rosell et al. BELIEF [37]	II	First	109	Erlotinib + bevacizumab	77	13.2 (95% CI: 10.3-15.5)	28.2 (95% CI: 21.4-41.8)
Stinchcombe et al. [38]	II	First	88	Erlotinib + bevacizumab *vs.* erlotinib	81 *vs.* 83, *P* = 0.81	17.9 *vs.*13.5, HR: 0.81; 95% CI: 0.50-1.31; *P* =0.39	32.4 *vs.* 50.6, HR: 1.41; 95% CI: 0.71-2.81; *P* = 0.33
Ichihara et al. Okoyama trial [39]	II	First	42	Gefitinib + bevacizumab	73.8 (95% CI: 58.0-86.1)	14.4 (95% CI:10.1-19.2)	n.r.
Kitigawa et al. [40]	II	First	16	Gefitinib-bevacizumab *vs.* gefitinib	44 *vs.* 50	5.4 (80% CI: 5.0-13.9) *vs.* 15.1 (80% CI: 6.2-23.5)	1-year OS: 0.667 (95% CI: 0.195-0.904) *vs.* 0.75 (95% CI: 0.315-0.931)
Hata et al. ABC trial [41]	II	Any line	32	Afatinib + bevacizumab	18.8 (95% CI: 7.2-36.4)	6.3 (95% CI: 3.9-8.7)	n.r.
Nakagawa et al. RELAY trial [42]	III	First line	449	Erlotinib + Ramucirumab *vs.* Erlotinib	76 *vs.* 75, *P* = 0.741	19.4 *vs.* 12.4 HR: 0.59; 95% CI: 0.46-0.76, *P* < 0.0001	n.r.

### Bevacizumab and erlotinib

Retrospective analyses in clinical trials conducted with erlotinib plus bevacizumab in unselected NSCLC patients suggested an advantage of the combination compared with erlotinib as a single agent in patients with EGFR activating mutations. On this basis, a number of phase II-III trials prospectively evaluated the efficacy of the combination in this setting of patients. In the first randomized phase II study (JO25567), Seto et al. [34], compared erlotinib plus bevacizumab *versus* erlotinib in 154 naïve Japanese NSCLC patients with EGFR activating mutations. Erlotinib plus *P* = 0.0015), but no OS (47 *versus* 47.4 months, HR 0.81, 95% CI: 0.53–1.23) [35]. A greater benefit from the combination was observed in patients with EGFR exon 19 deletion (median PFS 18 *versus* 10.3 months, HR 0.41, 95% CI: 0.24–0.72). On the contrary, in patients with EGFR point mutation L858R in exon 21, the benefit seemed to be modest and not clinically significant (HR 0.67, 95% CI: 0.38–1.18). Severe AEs were 91% with erlotinib and bevacizumab and 53% with erlotinib single agent. The most common AEs of any grade were rash, diarrhea, hypertension in the combination group; rash, diarrhea and paronychia in the erlotinib group. These findings were confirmed by the NEJ026 phase III study [36]. In this trial, 112 Japanese patients with EGFR-positive NSCLC were randomized to erlotinib plus bevacizumab and 114 to erlotinib single agent. The results from the preplanned interim analysis showed an improvement in PFS with the combination strategy compared with erlotinib single agent (16.9 compared with 13.3 months, HR 0.605, 95% CI: 0.417–0.877, *P* = 0.016), with a worse toxicity profile (grade 3–5 AEs in 88% *versus* 46% of patients, respectively). Moreover, erlotinib plus bevacizumab was associated with a higher frequency of hypertension (46% *versus* 10%), proteinuria (32% *versus* 5%), and non pulmonary haemorrhage (26% *versus* 3%). The BELIEF trial evaluated the activity of first-line erlotinib plus bevacizumab in patients with activating EGFR mutations, according to the expression of T790M resistance mutation [37]. Since the T790M mutation is linked to a high level of signal transducer and activator of transcription 3 (STAT3) protein which up-regulates the VEGF expression, this study was intended to be a proof of concept that adding bevacizumab to erlotinib could improve the treatment of de-novo T790M mutated patients. Among 109 patients treated with erlotinib and bevacizumab, 37 (34%) were T790M positive. The overall median PFS was 13.2 months (95% CI: 10.3–15.5), with a 12 month PFS survival rate of 55% (95% CI: 45–64). The primary end point was met only in T790M-positive patients, with a median PFS of 16 months and a 12 month PFS rate of 68%. Severe AEs were reported in 4.7% of patients included in the safety analysis (including one acute coronary syndrome and a colonic perforation). Inconclusive results were reported by a small phase II trial conducted in U.S. comparing erlotinib plus bevacizumab against erlotinib alone in 88 EGFR mutated patients (75% Caucasian) [38]. The combination failed to result in a significant difference in PFS (17.9 *versus* 13.5 months, respectively; *P* = 0.39) and OS (32.4 *versus* 50.6 months, respectively; *P* = 0.33). However, these results were affected by the small number of enrolled patients resulting in a low power of the study. The more frequent serious AEs in the combination and erlotinib arms were skin eruption (26% *versus* 16% of patients), diarrhea (9% *versus* 13% of patients), hypertension (40% *versus* 20% of patients), and proteinuria (12% *versus* 0% of patients).

### Bevacizumab and other EGFR TKIs

The Okoyama Lung Cancer Study Group 1001 evaluated the activity of the combination of gefitinib and bevacizumab in metastatic NSCLC patients harbouring the EGFR mutation [39]. Forty-two Japanese patients received gefitinib 250 mg/day plus bevacizumab 15 mg/kg every three weeks. Median PFS was 14.4 months and 1-year PFS rate was 56.7% (95% CI: 39.9–70.5), but the primary end point of the study was not met, because the lower limit of the CI reached the 40% threshold. Cutaneous rash, hypertension, proteinuria and diarrhea were the AEs more frequently reported. Negative results were also reported in another Japanese phase II trial that evaluated the activity of gefitinib plus bevacizumab in EGFR mutated patients with metastatic NSCLC cancer, without brain metastases and T790M expression, although the conclusions of this study were invalidated by the very small sample size (16 patients) [40]. A phase II trial (the ABC study) evaluated afatinib plus bevacizumab in metastatic EGFR mutated NSCLC, pretreated with erlotinib or gefitinib [41]. Thirty-two patients were enrolled in the trial; in 14 of them (44%) the T790M expression was detected. Six patients (18.8%, 95% CI: 7.2–36.4%) obtained an objective response. In patients T790M positive the ORR and median PFS were 14.3% and 6.3 months, while in T790M negative they were 22.2% and 7.1 months, respectively. AEs frequently reported were skin rash and diarrhea. Therefore, the combination seemed to be promising in T790M negative patients who were found to be resistant to other EGFR treatments.

### Ramucirumab and erlotinib

The Relay study compared ramucirumab plus erlotinib against erlotinib plus placebo in EGFR mutated metastatic NSCLC, without brain metastases [42]. The trial enrolled 449 patients with common EGFR mutations (patients with T790M mutation at baseline were excluded): 224 patients were randomized to erlotinib 150 mg/day plus ramucirumab 10 mg/kg every two weeks, while the remaining 225 patients were randomized to erlotinib plus placebo. The addition of ramucirumab to erlotinib improved PFS (19.4 *versus* 12.4 months, HR: 0.59, 95% CI: 0.46–0.76, *P* < 0.0001). OS data were immature. No difference in the percentage of patients who developed T790M mutation between the two groups was reported. One patient died during the treatment due to an event potentially related to the ramucirumab-erlotinib treatment. In the combination group, a serious AE was reported in 29% of patients, including hypertension (24%) and cutaneous rash (15%). In the control group, the 21% of patients had a serious AE, including hypertansaminasemia (8%) and cutaneous rash (9%). Recently, the combination of erlotinib and ramucirumab has been approved by FDA for the treatment of EGFR mutated naïve patients.

## Discussion and future perspectives

The combined treatment with angiogenesis and EGFR inhibitors showed contrasting results in patients with advanced NSCLC. In pretreated EGFR wild type patients, bevacizumab plus erlotinib improved RR and PFS in the BeTa trial [19], although the benefit was modest and did not translate into an OS advantage. Similarly, in the maintenance setting, erlotinib plus bevacizumab slightly improved PFS, but not OS. Moreover, the combination strategy was associated with more frequent AEs. In the first-line setting, bevacizumab plus erlotinib was even inferior to chemotherapy in EGFR wild-type patients [25–26], confirming the evidence coming also from the TORCH study that a strategy with an EGFR inhibitor is not recommended in unselected, naive, advanced NSCLC patients [43]. Negative results were also reported with the combination of oral VEGF inhibitors and erlotinib in the second-line treatment of advanced NSCLC patients with EGFR wild type tumors: in particular, sunitinib plus erlotinib resulted in greater responses and longer PFS, but this benefit was not clinically relevant and it was associated with more toxicity [32]. On the contrary, in EGFR mutated patients the combination of bevacizumab or ramucirumab with erlotinib showed a statistically significant and clinically relevant improvement of PFS [34, 36, 42] and this result led to the approval of these two regimens in the first-line therapy of patients with EGFR mutant NSCLC. No OS benefit was observed in the JO25567 trial [35], while the survival data are not mature for the two phase III trials with bevacizumab (NEJ026) and ramucirumab (RELAY). A meta-analysis of ten studies including 2, 802 participants with advanced NSCLC demonstrated that erlotinib plus bevacizumab failed to improve OS (95% CI: 0.87–1.12, *P* = 0.825), PFS (95% CI: 0.63–1.15, *P* = 0.297) and objective response rate (95% CI: 0.69–1.67, *P* = 0.758), and it had a worse toxicity profile (rash and diarrhea) [44]. However, a subgroup analysis suggested an improvement in overall survival (95% CI: 0.29–0.69, *P* < 0.001) for patients with EGFR mutations treated with combination therapy.

A possible explanation of the positive results of bevacizumab plus erlotinib in mutated EGFR patients and, conversely, the lack of a relevant effect of the same combination in EGFR wild type patients could be an improved delivery of erlotinib due to the effect of bevacizumab on tumor vessel physiology: the increased intratumoral concentration of the EGFR TKI could induce a positive effect only in oncogene addicted tumors, being non oncogene addicted tumors less sensitive to EGFR TKIs. However, other mechanisms might be involved in the synergism of anti-VEGF and anti-EGFR drugs. In fact, the EGFR system has been shown to promote the secretion of VEGF and other angiogenic factors in tumor cells [14–15]. Activation of the EGFR also induces the release of angiogenic factors, including VEGF, in mesenchymal stem cells within the tumor microenvironment [45]. Therefore, combination of VEGF and EGFR inhibitors can induce a more significant reduction of angiogenic factors within the tumor. Finally, pre-clinical studies also suggest a possible role of VEGF in the acquired resistance of cancer cells to anti-EGFR agents [46]. Further data on bevacizumab and erlotinib combination will be soon available from other ongoing clinical studies (Table 4). The Beverly study (ClinicalTrials.gov/NCT02633189) is the first phase III clinical trial that has enrolled Caucasian patients with naïve metastatic NSCLC and activating EGFR mutation [47]. Patients have been randomized in a 1:1 fashion in two arms: erlotinib plus bevacizumab or erlotinib alone. Co-primary end points are investigator and centrally assessed PFS. The trial is powered to detect an HR difference of 0.60 and it has completed the planned enrollment of 160 patients in 2018. Two further randomized phase II clinical trials are ongoing in Taiwanese (the Brilliant trial; ClinicalTrials.gov/NCT02655536) and Korean (the Ava-TA trial; ClinicalTrials. gov/NCT03126799) patients to compare bevacizumab and erlotinib combination *versus* erlotinib alone in metastatic naïve NSCLC patients harbouring EGFR mutations. Results of these two studies are soon to be released.

**Table 4.** Ongoing studies

**Trial**	**Phase**	**Setting**	**Stage**	**Patients**	**Treatment**	**Primary end points**
Beverly trial NCT02633189	III	Metastatic, first line	IV stage	200	Erlotinib + Bevacizumab *vs.* Erlotinib	PFS
Brilliant trial NCT02655536	II	Metastatic, first line	IV stage, brain metastases	109	Erlotinib + Bevacizumab *vs.* Erlotinib	PFS
Ava-Ta trial NCT03126799	II	Metastatic, first line	IV stage	128	Erlotinib + Bevacizumab *vs.* Bevacizumab	PFS
NCT02803203	I/II	Metastatic, first line	IV stage	50	Osimertinib + Bevacizumab	PFS
BOOSTER NCT03133546	II	Metastatic, second line	IV stage, T790M mutated	155	Osimertinib + Bevacizumab *vs.* Osimertinib	PFS
ACCRU NCT02971501	II	Metastatic, first line	IV stage, brain metastases	112	Osimertinib + Bevacizumab *vs.* Osimertinib	PFS
OWONBNSCLCLM NCT04148898	II	Metastatic, first line	IV stage, leptomeningeal metastases	80	Osimertinib + Bevacizumab *vs.* Osimertinib	ORR and iPFS
NCT04181060	III	Metastatic, first line	IV stage	300	Osimertinib + Bevacizumab *vs.* Osimertininib	PFS
NCT03909334	II	Metastatic, first line	IV stage, also in patients with brain metastases	150	Osimertinib + Ramucirumab *vs.* Osimertinib	PFS
NCT02789345	I	Metastatic, first line	IV stage, T790M mutated	74	Osimertinib + Ramucirumab + Necitumumab	DLTs

iPFS: intracranial progression-free survival

The major limitation of all these trials is that the control arm (erlotinib) is no longer the standard therapy for advanced NSCLC patients with activating EGFR mutations, due to the superiority of osimertinib over first generation EGFR TKIs demonstrated by the FLAURA trial [13]. On this basis, a number of clinical trials are currently evaluating the feasibility and activity of the combination of osimertinib with bevacizumab or ramucirumab (Table 4) in the first-line setting. A phase I/II trial is currently assessing the feasibility and efficacy of the combination of osimertinib and bevacizumab in EGFR mutated patients (ClinicalTrials.gov/NCT02803203). In the first part of the study, patients will receive bevacizumab 15 mg/ kg every three weeks plus osimertinib 40 mg or 80 mg daily; the maximum tolerated dose will be assessed and it will be used in the phase II part of the trial, which has PFS as primary outcome. Estimated study completion is planned for June 2020. A randomized phase II clinical trial (The Booster trial, ClinicalTrials. gov/NCT03133546) is also comparing the combination of bevacizumab and osimertinib against osimertinib alone as second-line therapy in patients already treated with an EGFR inhibitor and a confirmed T790M mutation; the primary outcome will be PFS, while the secondary outcomes will be ORR, OS and AEs; the trial will involve 25 Centers in Europe and Asia. Study completion is awaited for May 2022. A randomized phase II trial (ClinicalTrials.gov/NCT02971501) will determine the efficacy of first-line osimertinib plus bevacizumab against osimertinib alone in previously untreated EGFR mutated patients with brain metastases. Primary endpoint will be PFS, secondary endpoints will be OS, AEs, ORR, intracranial response rate and time to intracranial progression. Estimated study completion is awaited for July 2020. Another phase II study (ClinicalTrials.gov/NCT04148898) is evaluating the activity of bevacizumab plus osimertinib in EGFR mutated metastatic patients and leptomeningeal disease. Eligible patients will receive osimertinib or osimertinib and bevacizumab. Primary endpoints will be intracranial PFS and ORR; secondary end points will be leptomeningeal disease overall survival (intended to be the survival from the diagnosis of leptomeningeal disease), PFS and the appearance of AEs. Study completion is awaited on July 2021. Finally, a phase III trial (ClinicalTrials.gov/NCT04181060) will compare the efficacy of osimertinib plus bevacizumab against osimertinib alone in untreated metastatic NSCLC with EGFR mutation. Primary outcome measure will be PFS, secondary will be OS. The study will start in June 2020 and results are awaited in September 2025 and should definitively assess the efficacy of osimertinib and bevacizumab combination in naïve NSCLC patients with activating EGFR mutations. Two studies are evaluating the combination of ramucirumab with osimertinib. A phase II study (ClinicalTrials.gov/NCT03909334) will determine the activity of osimertinib plus ramucirumab against osimertinib in previously untreated EGFR mutated metastatic patients; primary endpoint will be PFS, secondary endpoints will be ORR, OS and AEs. Results are awaited for June 2024. A phase I trial (ClinicalTrials.gov/NCT027893459) will assess the safety of ramucirumab or necitumumab plus osimertinib in advanced NSCLC patients with EGFR activating mutations and expression of T790M, in progression after EGFR TKIs. The primary end point will be DLTs; among secondary end points there are PFS and OS; results are awaited for May 2020.

However, despite the relevant benefit in PFS observed in clinical trials, several issues may limit the use of the combinations of angiogenesis inhibitors plus EGFR TKIs in EGFR mutant patients: the need of an intravenous infusion of bevacizumab or ramucirumab with consequent frequent patients admissions in Hospital; the increased incidence of treatment associated AEs compared with EGFR TKIs as single agents; the exclusion of patients with tumors located in central position or at risk of hemorrhage; the frequent exclusion of patients with brain metastases; the increased costs of the combination. The identification of molecular biomarkers that could predict the response to the combination of angiogenesis and EGFR inhibitors, identifying patients which could derive more benefit from this combination, is an important goal of research to optimize the combined use of these agents.

## Abbreviations

AE: adverse event
bFGF: beta fibroblast growth factor
CI: confidence interval
DLT: dose limiting toxicity
EB: erlotinib plus bevacizumab
EGFR: epidermal growth factor receptor
FGFR: fibroblast growth factor receptors
Flt-4: FMS-related tyrosine kinase 4
HER 1: human epidermal growth factor receptor 1
HR: hazard ratio
iPFS: intracranial progression-free survival
KIT: stem cell factor receptor
KRAS: kirsten rat sarcoma
NPR: non progression rate
NSCLC: non-small-cell lung cancer
ORR: overall response rate
OS: overall survival
PDGFR: platelet-derived growth factor receptors
PFS: progression free survival
PGB: cisplatin, gemcitabine and bevacizumab
PR: partial response
RAF: rapidly accelerated fibrosarcoma
RET: rearranged during transfection
RR: response rate
STAT3: signal transducer and activator of transcription 3
TKI: tyrosine kinase inhibitor
VEGF: vascular endothelial growth factor
VEGFR: vascular endothelial growth factor receptor
WT: wild type

## Declarations

### Copyright

© The Author(s) 2020.
